# Supplementary material for: Coordinated control of adiposity and growth by anti‐anabolic kinase ERK7
Source: EMBO Rep. 2020 Dec 28;22(2):e49602. doi: 10.15252/embr.201949602 (PMC7857433; doi:10.15252/embr.201949602)
Supplement: Supplementary file 1 — Expanded View Figures PDF [file EMBR-22-e49602-s001.pdf]

Expanded View Figures

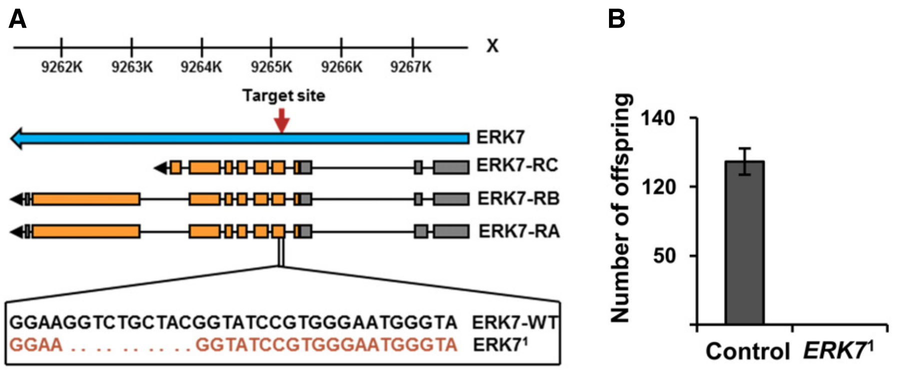

Figure EV1. ***ERK7[1]* mutant males are infertile.**

A Detailed presentation of the *ERK7[1]* mutation.

B Fertility assay for control and *ERK7[1]* male flies. $N = 4$ replicates of 10 male and 10 female flies/replicate for each genotype. Error bars display standard deviation (SD).

Data information: N stands for the number of biological replicates. Error bars display standard deviation (SD). $*P < 0.05$, $**P < 0.01$, $***P < 0.001$ (Student's *t*-test).

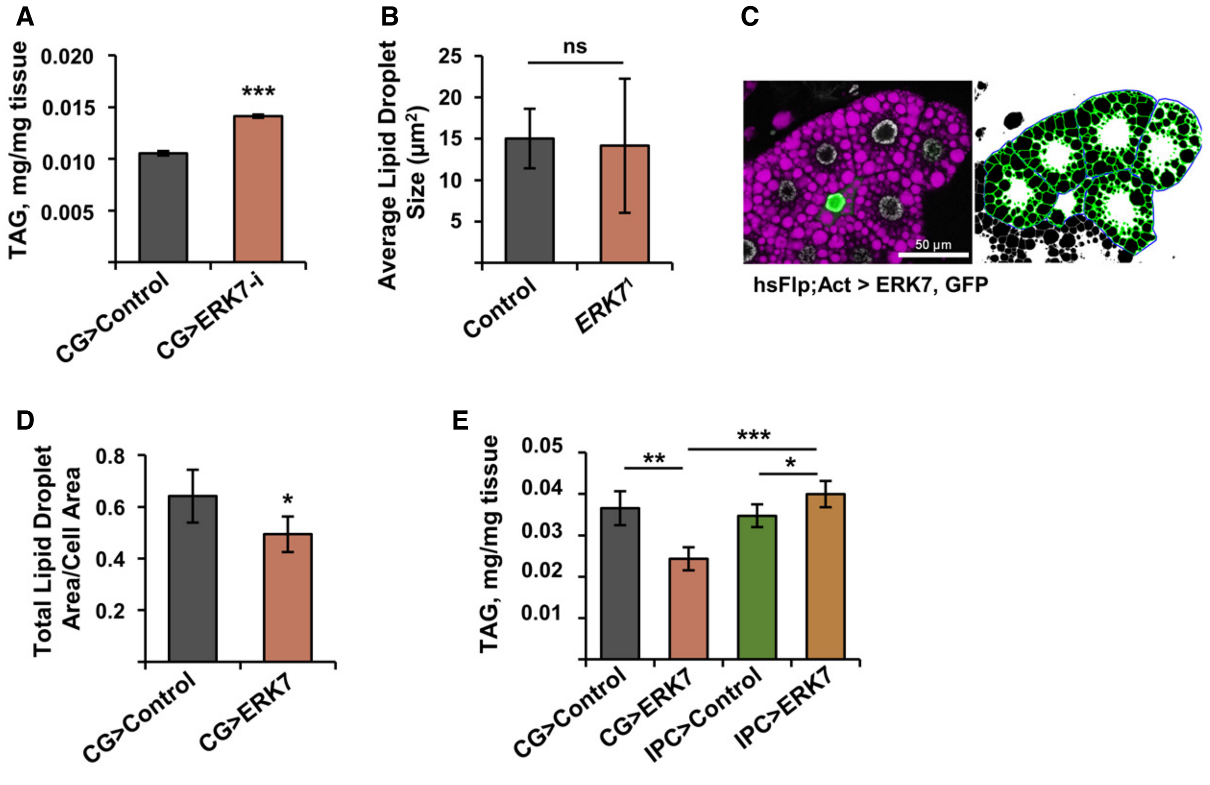

Figure EV2. ERK7 inhibits lipid storage.

A Knockdown of ERK7 by independent RNAi (VDRC v109661) leads to elevated larval triacylglycerol (TAG) levels ($N = 3$ replicates of ≥ 10 larvae/replicate for each genotype).

B Average lipid droplet size is unchanged in *ERK7[1]* mutants ($N = 30$).

C Representative image of lipid droplet quantification, using FIJI. Purple: LipidTOX, green: GFP.

D Total area of lipid droplets in ERK7 overexpressing clones, when normalized by cell area ($N \geq 4$ cells per genotype).

E In contrast to CG>ERK7, ERK7 expression in the IPCs does not decrease organismal TAG levels ($N = 4$ replicates of ≥ 10 larvae/replicate for each genotype).

Data information: N stands for the number of biological replicates. Error bars display standard deviation (SD). ns: not significant, $*P < 0.05$, $**P < 0.01$, $***P < 0.001$ (Student's *t*-test).

© 2021 The Authors

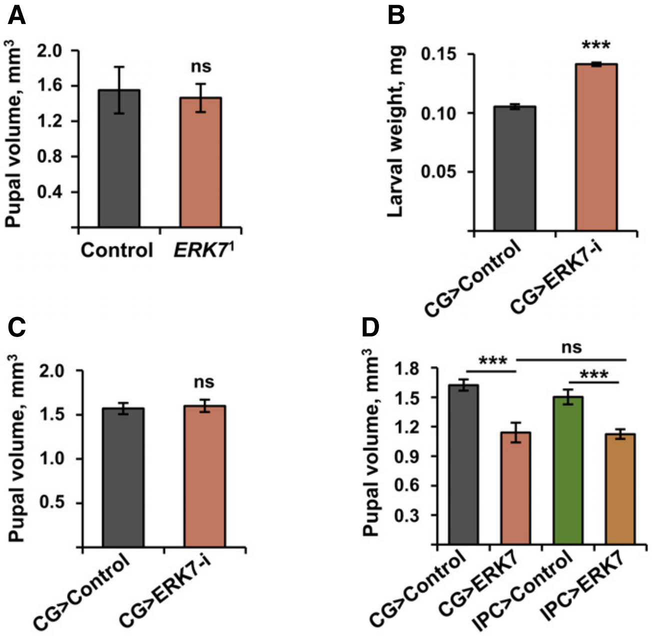

Figure EV3. ERK7 inhibits growth.

A Pupal volumes of control and *ERK7*^{1} mutant animals (N = 4 replicates of 10 pupae/replicate for each genotype).

B Knockdown of ERK7 by independent RNAi (VDRC v109661) leads increased larval weight at 72 h AED (N = 3 replicates of ≥ 10 larvae/replicate for each genotype).

C Fat body-specific knockdown of ERK7 by RNAi (BDSC 56939) does not impact on pupal volume (N = 6 replicates of 10 pupae/replicate for each genotype).

D Ectopic ERK7 expression in the fat body (CG-Gal4) or IPCs (dILP2-Gal4, UAS-GFP) leads to comparable reduction in pupal volume (N = 4 replicates of 10 pupae/replicate for each genotype).

Data information: N stands for the number of biological replicates. Error bars display standard deviation (SD). ns: not significant, ***P < 0.001 (Student's t-test).

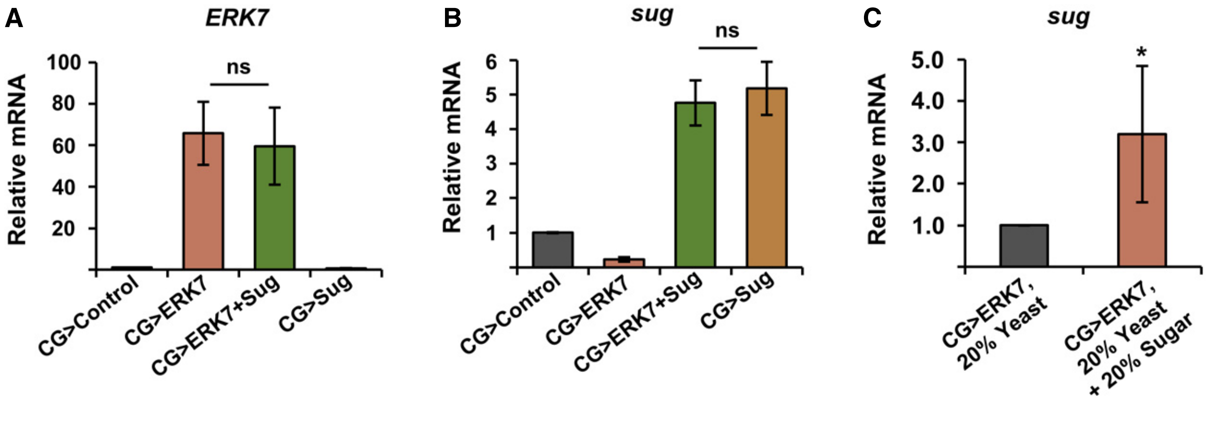

Figure EV4. *ERK7* and *sugarbabe* expression controls.

A, B Simultaneous expression of two transgenes (*ERK7* and *sugarbabe*) does not reduce the expression levels when compared to single transgene expression by *CG-GAL4*. mRNA expression analysis of *ERK7* and *sugarbabe* from fat bodies using qRT–PCR (N = 4 replicates of 10 fat bodies/replicate for each genotype). Expression of RP49 was used for normalization.

C *sugarbabe* expression in CG>ERK7 fat bodies is elevated upon sugar feeding. mRNA expression analysis of *sugarbabe* in CG>ERK7 fat bodies by qRT–PCR. Expression of RP49 was used for normalization (N = 4 replicates of 10 fat bodies/replicate for each genotype and diet).

Data information: N stands for the number of biological replicates. Error bars display standard deviation (SD). ns: not significant, *P < 0.05 (Student's t-test).

© 2021 The Authors
